# Epidemiology and control strategies for tuberculosis in countries with the largest prison populations

**DOI:** 10.1590/0037-8682-0060-2022

**Published:** 2022-11-21

**Authors:** Caroline Busatto, Dienefer Venske Bierhals, Julia Silveira Vianna, Pedro Eduardo Almeida da Silva, Lia Gonçalves Possuelo, Ivy Bastos Ramis

**Affiliations:** 1 Universidade Federal do Rio Grande, Programa de Pós-Graduação em Ciências da Saúde, Rio Grande, RS, Brasil.; 2 Universidade de Santa Cruz do Sul, Programa de Pós-Graduação em Promoção da Saúde, Santa Cruz do Sul, RS, Brasil.

**Keywords:** Mycobacterium tuberculosis, Health, Prisoners

## Abstract

Tuberculosis (TB) is a serious infectious disease, and its control is considered a challenge, especially among vulnerable populations such as prisoners. The occurrence of TB in prisons is an alarming public health problem in many countries. This integrative review aims to describe the epidemiology of TB and control strategies for this disease in countries with the largest prison populations. Studies have shown that it is essential to know the prevalence of TB in prisons of each country. This is because it can serve as an indication of the need for action in prisons to reduce TB rates, including improving the structure of prison environments, rapidly and accurately diagnosing new cases, identifying drug-resistant strains, and implementing effective and directly observed treatment for TB.

## INTRODUCTION

Tuberculosis (TB) remains a communicable disease that causes most deaths worldwide, even in times of the COVID-19 pandemic, affecting various age groups and social classes^1^ ^,^ ^2^. In this sense, there are populations with an increased risk of developing TB (called key populations by the Global Fund to Fight AIDS, Tuberculosis, and Malaria), such as people living with human immunodeficiency virus (HIV), migrants, refugees, children, diabetic individuals, indigenous populations and prisoners^3^ ^,^ ^4^. 

Prisons are propitious environments for the transmission of *Mycobacterium tuberculosis,* since in most of these settings, there is a high turnover of prisoners, in addition to the overcrowded and poorly ventilated space^5^. The frequent transfer of prisoners between prisons creates a network of prison contacts that likely facilitates the spread of *M. tuberculosis* and can spread the risk of infection to the general population^6^. 

The estimated prevalence of latent tuberculosis infection (LTBI) and active TB disease in prisons is higher than that in the general population. In addition, many of these individuals use tobacco, alcohol, and illicit drugs, and are thus considered risk factors, as they favor the development of active TB. Unfortunately, measures to control TB in these settings are lacking, and this population has limited access to health care^7^ ^-^ ^9^.

It is important to highlight that more than 10.8 million people will be held in prisons worldwide in 2021. The United States of America (USA) has the world's highest index, with more than 2 million prisoners, followed by China (1.7 million), Brazil (811,000), India (478,000), the Russian Federation (469,000), Turkey (291,000), Thailand (285,000), Indonesia (270,000), Mexico (222,000), Iran (189,000) and the Philippines (165,000)^10^ ^,^ ^11^. In this context, using the current list of countries with the largest prison populations, this integrative review aimed to describe the epidemiology of TB and strategies to control this disease in these countries.

## METHODS

This integrative review defined the guiding question as “What is the epidemiology of TB and what actions are carried out to control the disease in countries with a higher prison load?” The search and selection of articles were carried out between October and November 2021 in the following databases: Medical Literature Analysis and Retrieval System Online (Medline), Latin American and Caribbean Literature in Health Sciences (Lilacs), Virtual Scientific Electronic Library Online (SciELO), PubMed, and Google Scholar. The descriptors chosen for the development of this research were “Tuberculosis”, “Prisons”, “Inmates”, “Prisoners” and the name of the countries with a higher TB prison burden (total prison population), described in the World Prison Population List^10^. There was no restriction on the date of publication, and regarding inclusion criteria, original articles written in English, Spanish or Portuguese that addressed “Tuberculosis” and “Prisons” as a central theme were eligible for inclusion. 

Out of more than 5,000 publications identified during the initial search, 200 articles met the inclusion criteria and provided information on the central theme. The analysis was performed critically, seeking explanations for the different results of the studies, first through the reading of abstracts, and later, the reading of the complete article, conducted in pairs. In case of discrepancies, a third researcher was consulted. Regarding data collection, detailed information was extracted from each study, such as objectives, interventions carried out in the study scenario, and evidence of the results. We describe the epidemiology of TB based on the prevalence and incidence reported in studies conducted in these countries. Data synthesis was performed by comparing theoretical knowledge and identifying conclusions and implications that resulted in the integrative review^12^.

## RESULTS AND DISCUSSION

### Epidemiology of active TB

Overlooking TB prevention and control, especially in regions with high incarceration rates, can have serious consequences for the control of TB in both prisoners and the general population. It is fundamental to know the prevalence of TB in prisons in each country, as this may indicate the need to adopt measures to control the disease in these high-risk environments^13^ ^-^ ^15^. 

**FIGURE 1:**
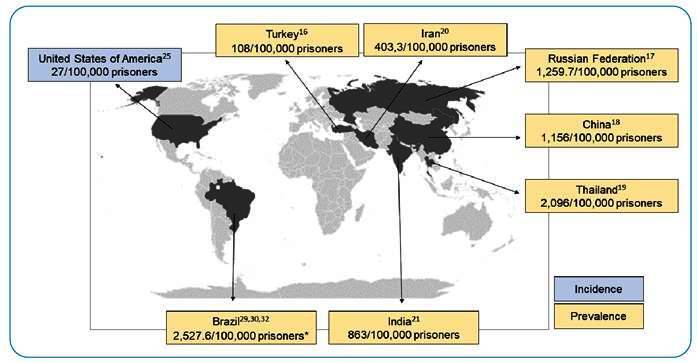
Geographical representation of the prevalence and incidence of *Mycobacterium tuberculosis* among prisoners from countries with the largest prison populations.

Between 2006 and 2007, active TB cases were tracked in 10 prisons located in Turkey, with a prevalence of 108/100,000 prisoners. The authors pointed out that all TB cases occurred in the same prison, which is the largest and most crowded area of the country. After this result, the conditions of this prison establishment were reviewed, and the cells that accommodated 50 or more prisoners began to hold a maximum of three people (Figure 1)^16^. 

In 2021, Dadu *et al*. presented a descriptive study on the incidence of active TB in prisons in Europe, and the main finding was a gradual decrease in cases between 2014 and 2018. In the Russian Federation, a decrease in the incidence of TB was observed from 1,683.6/100,000 to 1,259.7/100,000 prisoners between 2014 and 2018^17^. This finding may also be related to TB control interventions carried out by national governments through strategies launched by the World Health Organization (WHO) in 2014^13^.

Some Asian countries have the highest population detention index and a higher prevalence of TB among prisoners. In 2019, the prevalence of active TB (1,156/100, 000) in a prison in China was significantly higher than that in the general population (514/100,000)^18^. In Thailand prisons, an active TB prevalence of 2,096/100,000 prisoners was found, an alarming number when compared to the prevalence found in the general population (149/100,000)^19^. In Iran, there are few studies with active TB data in prisons; however, in 2014, a study found a TB prevalence of 403.3/100,000, when the annual rate of cases in the general population during this period was 16.4/100,000^20^. A study conducted in southern India in 2019 showed a prevalence of 863/100,000 prisoners, a rate 2.5 times higher than that in the general population^21^. In Indonesia, prisoners who had been detained previously had a 2.3 times greater risk of developing TB than those who had not been in prisons before^22^.

In America, most specifically USA, the goal of eliminating TB through a strategy to quickly identify and treat cases, and assess exposed contacts to limit secondary cases resulting from the disease was effective in reducing active TB cases^23^ ^,^ ^24^. In California, a study conducted in 2017 showed that the incidence of active TB among prisoners decreased significantly, with rates ranging from 91/100,000 in 1995 to 27/100,000 in 2013; however, prisoners still have TB incidence rates three to four times higher than the general population^25^.

Moreover, improving investigations into the contact of patients at risk of developing TB among countries neighboring the USA, such as Mexico, likely resulted in a decrease in the number of TB cases in both countries^26^. Mexico is a country with endemic TB, with more than 20,000 cases reported annually^27^. In 2012, a cohort study was carried out among HIV-infected prisoners in Mexico City, where an active TB prevalence of 10.1/100,000 prisoners was observed, highlighting that the use of drugs, alcohol, and malnutrition were the most reported risk factors among the study participants^28^.

While investigating the incidence of TB among incarcerated populations across all WHO geographic regions, it was found that across America, the incidence was substantially higher in South America (970/100,000 prisoners) than in North America (30/100,000 prisoners)^15^. According to Walter *et al.* (2021), the increase in TB case trends in South America is driven by Brazil, this country was responsible for 45% of the increase in TB cases among prisoners in the region, from 2011 to 2017. The disease burden attributable to prisons is growing owing to rising incarceration rates^5^. 

In Brazil, Valença *et al*. (2015) reported an even higher prevalence of 4,712/100,000 prisoners in Pelotas City, in an active and passive screening study^29^. In 2019, with active screening of active TB cases in the Central Prison of Porto Alegre, the capital of Rio Grande do Sul, the prevalence of TB was 1,898/100,000 prisoners^30^. The state of São Paulo has the largest prison population in Brazil; a study carried out in this region observed the high annual incidence of TB in the prison system from 927.5/100,000 prisoners in 2015, to 1,232.6/100,000 prisoners in 2017^31^. In comparison, in 2018, in Mato Grosso do Sul state, the active TB prevalence in prisoners was 972.9/100,000, and information was obtained from 35 prisons in the region^32^.

From the data presented, it was observed that the expansion of incarceration in recent decades is related to the high burden of TB in prisons. We found that incidence and prevalence data vary between countries, which may be related to the diagnostic criteria used in each study. In general, the most commonly used diagnostic methods among the articles covered in this review were radiography, smear microscopy, and mycobacterial culture.

It is important to highlight that, for many prisoners, the prison environment is one of the few places where they can obtain information and guidance on health care, mainly because many come from a marginalized population with little social support. Thus, prisons with an organized health service can promote health through the early detection and treatment of diseases such as TB, thus minimizing the impact of infectious diseases on the general population^33^.

### Epidemiology of latent tuberculosis infection (LTBI)

Another concern of prison health agencies is the high rates of LTBI. The LTBI is defined as a state of persistent immune response to stimulation by *M. tuberculosis* antigens without evidence of clinically manifested active TB^2^. The magnitude of LTBI in the prison system is poorly understood; however, some studies have reported high rates of LTBI. This is the case for prisons in China, in 2016, with an LTBI rate above 50%, which is higher than that of the general population (13.5-24.3%), using an interferon-γ release assay (IGRA)^34^. From August 2018-November 2019, Gatechompol *et al.* (2021) conducted a study in a maximum-security prison in Thailand, with 46.5% of patients testing positive for LTBI using tuberculin skin test (TST) and IGRA^35^.

In Brazil, in 2013, a study carried out in two prisons in Minas Gerais reported a rate of 25.2% LTBI by TST, which was associated with self-reported contact with individuals having active TB and the use of inhaled drugs^36^. Two prisons in São Paulo in 2008, using TST, reported a rate of 73% LTBI^37^. On the other hand, in the USA, an LTBI rate of 6.3% among prisoners was detected between 2011 and 2013 using IGRA, which was relatively low compared to the data presented above; however, these data were consistent with the low incidence of active TB in the country^38^. 

Moreover, we highlight a study in Iran that was planned in two stages in 2013. First, researchers determined the prevalence of LTBI among prisoners using the TST. In the second stage, after six months, prisoners with a negative TST at the first visit were invited to undergo a second TST to determine the incidence of LTBI. They obtained a prevalence rate of 62.6% for LTBI and an incidence rate of 7.6% for LTBI^39^. From the presented data, we emphasize that there is a high burden of LTBI in prisons. However, there is a lack of studies with recent data on the prevalence and incidence of TB in countries with high TB burden in prisons. Research with this focus is important for implementing prevention and control measures for TB, as this population is at a high risk of developing the disease^40^.

### Prevention and control measures for TB

TB was included in Goal 3.3 of the Sustainable Development Goals by the United Nations, which aimed to end epidemics of AIDS, TB, malaria, and neglected tropical diseases by 2030^41^. Many strategies have been established to achieve this goal, including the DOT Strategy (Directly Observed Therapy), Stop TB Strategy and End TB Strategy^42^. Prisons are environments that directly influence the burden of TB^43^; therefore, basic health care improvements are needed in prisons, such as the implementation of preventive and educational measures, improved ventilation, and reduction in the number of prisoners per cell (Table 1).

**TABLE 1:** Primary TB control measures to decrease the burden of TB in prisons.

Author	Place	Main results
Johnstone-Robertson *et al.,* 2011^54^	South Africa	Cross ventilation: Suggested not to close the cell windows at night and to use barred doors instead of solid doors in cells, allowing better ventilation.
Santos *et al.,* 2012^57^	Brazil	Architectural solutions: Improve lighting and ventilation conditions with openings on opposite walls and ventilation on two levels on the same wall or roof.
Brazil, 2014^70^	Brazil	Prison Primary Health Care Team: Implementation of prison health teams, including doctors, nurses, and dentists.
Sander *et al.,* 2017^49^	Global	Harm reduction: Opioid substitution therapy, needle and syringe programs, overdose and withdrawal prevention, and HIV and HCV testing and treatment.
Katyal *et al.,* 2018^38^	United States of America	Latent tuberculosis infection detection: The QuantiFERON®-TB Gold In-Tube (QFT-GIT) IGRA test was implemented at a women's prison and at an all-men’s prison.
Adane *et al.,* 2019^51^	Ethiopia	Peer education: Prisoners receive training in identifying TB cases and then provide health education to other prisoners.
Mabud *et al.,* 2019^59^	Brazil	Annual mass screening for TB: Identification of symptoms suggestive of the disease and diagnosis of TB.
Sousa *et al.,* 2020^52^	Brazil	TB Hotspot DetectOR: Collection of aerosols from environment allowing the identification of *M. tuberculosis* in the cough of prisoners.
dos Santos *et al*., 2022^64^	Brazil	Pooling Sputum Samples: Tested prisoner sputum combinations with GeneXpert MTB/RIF Ultra, allowing for more frequent triage of cases, resulting in cost containment.

In addition, specific groups present an increased risk of *M. tuberculosis* infection and its progression to active TB. These include those infected with HIV, hepatitis virus, and *Treponema pallidum*. Lifestyle also influences the development of these diseases. Therefore, in the prison environment, this issue is aggravated, as prisoners exhibit risky behaviors, such as the use of alcohol, drugs, and tobacco, in addition to unprotected sex and sharing of syringes and razor blades. For example, in prisons in Iran and Thailand, injection drug use has resulted in large outbreaks of HIV; in the USA, HIV prevalence among prisoners is three times higher than that in the general population. At the same time, approximately 33% of American prisoners live with hepatitis C (HCV)^9^ ^,^ ^44^ ^-^ ^46^.

Therefore, prisons are important locations for the development and implementation of public health interventions. Among these interventions are harm reduction, which includes opioid substitution therapy, needle and syringe programs (NSPs), overdose and withdrawal prevention, and HIV and HCV testing and treatment. In USA, the implementation of NSPs has resulted in a decrease in new HIV infections among prisoners using injectable drugs. The implementation of harm reduction services also proved extremely cost-effective, where it was estimated that every dollar invested in NSPs returned $4 in health savings^47^ ^-^ ^50^.

Peer education was another strategy. This strategy was used to educate prisoners in Ethiopia about health issues and has already proven to be an important means of improving TB case detection. The prisoners were recruited and received a 3-day training course to identify presumptive TB cases, after which they provided education to all other prisoners about TB prevention and control every two weeks for one year. This method led to an improvement in the rate of case detection, a decrease in the duration of symptoms before treatment, and an increase in treatment success, which consequently decreased the bacillus transmission. Therefore, this is a great alternative, particularly for prisons in low-income countries^51^.

Regarding innovative strategies, TB Hotspot DetectOR (THOR) collects aerosols carrying microspheres, *Bacillus globigii* spores, and *Mycobacterium bovis* BCG, concentrating these microparticles onto a collector piece designed for subsequent detection analysis. The unit was successfully operated in the complex environment of a prison hotspot, allowing the detection of a molecular signature of *M. tuberculosis* in a prisoner’s cough. Therefore, the implantation of this electrostatic air sampler device can lead to a measurable impact in the detection of TB cases by screening individuals through the aerosols that they generate^52^.

Regarding the structure of prisons, the recommendation is a minimum allocation of 5.4 m^2^ of space per prisoner. However, prisoners are usually in overcrowded cells for extended periods, with a space of less than 2.1 m^2^ per prisoner. In addition, cells have little ventilation; therefore, limiting the number of people is not sufficient to reduce the risk of transmission of *M. tuberculosis* ^32^ ^,^ ^53^ ^,^ ^54^. Furthermore, Verma *et al.* (2022) conducted a study in two prisons with a high burden of TB in Brazil and found that *M. tuberculosis* can often be detected in environments recently occupied by individuals with active TB. Through environmental surface swab collection, this methodology can serve as a tool for characterizing the risk of exposure^55^.

In this sense, a study in Brazil showed, that the optimization of cell ventilation can lead to a decrease in TB cases. Improving ventilation would slow the secondary infection rate and provide a greater window for diagnosing a case before most cellmates are infected^56^. Another study in Brazil proposed architectural solutions for the improvement of lighting and ventilation conditions so that they could be incorporated into the construction of new prisons and renovation of current prisons. One of these solutions would be to allow air flow in the cells through openings in opposite walls and ventilation at two levels on the same wall or roof^57^. In a South African prison, it was suggested that cell windows should not be closed at night, and barred doors should be used instead of solid doors in the cells, allowing for improved cross-ventilation. In general, natural ventilation systems are generally low-cost compared with mechanical ventilation and may be more viable in environments with limited resources^54^.

Other essential components of TB control include LTBI detection and prison entry screening programs^58^. There are recent examples of LTBI screening programs in prisons in the USA and China. To improve the coverage of LTBI screening in US prisons, the QuantiFERON®-TB Gold In-Tube (QFT-GIT) IGRA test was implemented at a women's prison in early 2011 and at an all-men’s prison by 2012. However, among countries with a high TB burden in prisons, such as Brazil, Iran, and Mexico, there is no official strategy for detecting prisoners with LTBI or active TB before they enter or while they are in prison^36^ ^-^ ^39^.

Regarding active TB in prisons, a study carried out in Brazil showed that an annual mass screening for TB within prisons would reduce the incidence of TB in prisons by 47.4%^59^. The strategies used for detection include the identification of symptoms suggestive of the disease, chest radiography, and laboratory diagnostics^60^ based on microscopy, culture, and/or the GeneXpert MTB/RIF assay (Cepheid Inc., Sunnyvale, CA, USA). This molecular test was recommended and approved by the World Health Organization (WHO) in 2012. The GeneXpert MTB/RIF detects *M. tuberculosis* and the main mutations related to rifampicin (RIF) resistance in less than two hours^61^ ^,^ ^62^. This is a very important approach because it allows for rapid and accurate TB diagnosis, as well as detection of RIF resistance, one of the main drugs used in the treatment of the disease. It is important to highlight that in the Russian Federation, screening with GeneXpert MTB/RIF, in comparison with chest radiography, reduced the overall TB prevalence (from 2.8% to 2.3%) in prisoners and drug resistance prevalence (from 0.7% to 0.6%)^63^.

Another rapid molecular diagnostic alternative using GeneXpert MTB/RIF is the GeneXpert MTB/RIF ultra-sputum pooling, which can be a sensitive and efficient approach to mass screening in prisons. In a study in Brazil, a test was performed by selecting combinations of 4, 8, 12, and 16 sputum samples from prisoners, which were then tested using Ultra, in addition to calculating the costs of this grouping. Therefore, in settings with a higher TB prevalence, combinations of four and eight samples were more efficient than larger combinations and led to greater cost savings^64^.

Unfortunately, in some regions of the world, such as Asia and America, challenges persist in the diagnosis and treatment of TB. In a Northeast Indian prison, prisoners with suspected TB are referred to specialized TB centers, which are 10-30 km from the prison^65^. In another 157 prisons in India, TB diagnosis and treatment services were available in only 18% and 54% of prisons, respectively. Therefore, it is necessary to strengthen the entry screening, diagnostic, and treatment services in all prisons to contain the TB burden in this high-risk group and disseminate this information through publications, which are still scarce on this topic^66^. In contrast, in a prison in Brazil, a TB diagnosis center was introduced in 2010. Entrance screening for TB included symptom tracking and chest radiography in all patients. Prisoners with symptoms or abnormal chest radiographs underwent microscopy, culture, and drug susceptibility tests. In 2014, Brazil's National Tuberculosis Program strengthened the TB control in this prison by introducing the GeneXpert MTB/RIF assay into the diagnostic routine^30^.

However, detection alone is not sufficient for TB control, and it is necessary to correctly treat diagnosed patients. TB is a curable disease in practically all cases if treatment is performed adequately. Therefore, it is recommended that the treatment of TB in prisoners be performed using DOT. In this strategy, health professionals must strictly monitor drug intake to strengthen the bond between patients and professionals^67^ ^,^ ^68^.

In 2019, Allgayer *et al.* analyzed the care and surveillance actions related to TB, such as the use of DOT, in 13 prisons in the southern region of Brazil and observed that 53.8% of the establishments used DOT for all patients, and 15.4% used DOT only when there was no adherence to treatment by the patient^69^. It is important to consider the DOT strategy for all patients and endeavor to cure them. In addition, strengthening the communication strategy between intra- and extramural health facilities when prisoners are released before the TB treatment ends, is essential. 

Some limitations found in the construction of this integrative review were the lack of studies related to some themes and the lack of deepening of the subjects (such as incidence and prevalence) in the countries covered. Furthermore, while some of these strategies require further studies for future implementation, other simpler actions can already be taken, such as the expansion of access to the health system and continuing health education for workers in the prison system. Therefore, prisons are important sites for the development and implementation of public health interventions aimed at reducing the incidence of infectious diseases. An example is a Brazilian health policy called the “Prison Primary Health Care Team,” which establishes the implementation of prison health teams, including doctors, nurses, nursing technicians, dentists, oral health assistants, psychologists, social workers, and occupational therapists, aiming to expand actions/interventions for TB control among prisoners^9^ ^,^ ^70^ ^,^ ^71^.

An articulated set of actions is required in prisons, including improvement of the structure of prison environments, rapid and accurate diagnostic methods for the detection of new cases and drug resistance, effective and directly observed treatment, and appropriate preventive measures such as vaccination and treatment of LTBI^72^. Finally, it is of great importance to encourage interest in the prison health in a humanitarian manner, with care to promote and protect the health of prisoners and healthcare and safety workers.
